# Identification of key genes involved in nasopharyngeal carcinoma^☆^

**DOI:** 10.1016/j.bjorl.2016.09.003

**Published:** 2016-09-26

**Authors:** Xue Jiang, Lichun Feng, Baoqiang Dai, Liping Li, Weiwei Lu

**Affiliations:** Cangzhou Central Hospital, Department of Otorhinolaryngology, Cangzhou, Hebei, China

**Keywords:** Nasopharyngeal carcinoma, Differentially expressed genes, Enrichment analysis, Regulatory network, Carcinoma nasofaríngeo, Genes diferencialmente expressos, Análise de enriquecimento, Rede reguladora

## Abstract

**Introduction:**

Nasopharyngeal carcinoma is the most common cancer originating from the nasopharynx.

**Objective:**

To study the mechanisms of nasopharyngeal carcinoma, we analyzed GSE12452 microarray data.

**Methods:**

GSE12452 was downloaded from the Gene Expression Omnibus database and included 31 nasopharyngeal carcinoma samples and 10 normal nasopharyngeal tissue samples. The differentially expressed genes were screened by ANOVA in the PGS package. Using the BiNGO plugin in Cytoscape and pathway enrichment analysis in the PGS package, functional and pathway enrichment analyses were performed separately to predict potential functions of the differentially expressed genes. Furthermore, Transcription factor-differentially expressed gene pairs were searched, and then the transcription factor-differentially expressed gene regulatory network was visualized using Cytoscape software.

**Results:**

A total of 487 genes were screened as differentially expressed genes between the nasopharyngeal carcinoma samples and the normal nasopharyngeal tissue samples. Enrichment analysis indicated that PTGS2 was involved in the regulation of biological process and small cell lung cancer. ZIC2 and OVOL1 may function in nasopharyngeal carcinoma through targeting significantly up-regulated genes (such as PTGS2, FN1, CXCL9 and CXCL10) in the Transcription factor-differentially expressed gene regulatory network (e.g., ZIC2→PTGS2 and OVOL1→CXCL10).

**Conclusion:**

PTGS2, FN1, CXCL9, CXCL10, ZIC2 and OVOL1 might play roles in nasopharyngeal carcinoma.

## Resumo

**Introdução:**

O carcinoma nasofaríngeo é o câncer mais comum originário da nasofaringe.

**Objetivo:**

Estudar os mecanismos do câncer de nasofaringe; dados do microarray GSE12452 foram analisados.

**Método:**

GSE12452 foi obtido da base de dados Gene Expression Omnibus e inclui 31 amostras de carcinoma nasofaríngeo e 10 amostras de tecido nasofaríngeo normal. Os genes diferencialmente expressos foram analisados por ANOVA no kit PGS. Usando o plugin BiNGO no Cytoscape e análise de enriquecimento da via no kit PGS, análises de enriquecimento funcional e da via foram realizadas separadamente para prever as potenciais funções dos genes diferencialmente expressos. Além disso, os pares Fator de Transcrição - genes diferencialmente expressos foram pesquisados e em seguida a sua rede reguladora foi visualizada usando o programa Cytoscape.

**Resultados:**

Um total de 487 genes foram analisados como genes diferencialmente expressos entre as amostras de carcinoma nasofaríngeo e amostras de tecido nasofaríngeo normal. A análise de enriquecimento indicou que PTGS2 estava envolvido na regulação do processo biológico e câncer pulmonar de pequenas células. ZIC2 e OVOL1 podem funcionar no carcinoma nasofaríngeo almejando-se de maneira significativa os genes suprarregulados (como o PTGS2, FN1, CXCL9 e CXCL10) na rede reguladora de fator de transcrição - genes diferencialmente expressos (p.ex., ZIC2→PTGS2 e OVOL1→CXCL10).

**Conclusão:**

PTGS2, FN1, CXCL9, CXCL10, ZIC2 e OVOL1 podem desempenhar alguns papéis no carcinoma nasofaríngeo.

## Introduction

As the most common cancer originating from the nasopharynx, nasopharyngeal carcinoma (NPC) caused approximately 86,700 new cases and 50,800 deaths globally in 2012.^1^ NPC is extremely common in Southeast Asia and southern China, with more than 50,000 new cases each year.^2^, ^3^ NPC can be induced by multiple factors including heredity, viral factors and environmental influences.^4^ Most cases of NPC are correlated with Epstein–Barr Virus (EBV) infection, which is a B-lymphotropic herpesvirus possessing growth-transforming properties.^5^ Therefore, it is of great importance to study the mechanisms of NPC.

Many studies investigating the mechanisms of NPC have been published. There are several genes (such as C-myc, AKT1, p53, MDM2, LMP1 and PTEN) implicated in the pathogenesis of NPC because they are often amplified or altered in patients with this disease.^6^, ^7^, ^8^ Disabled 2 (DAB2) is frequently down-regulated by promoter hypermethylation and may be a potential tumor suppressor in NPC.^9^ Previous studies show that the potential tumor suppressor gene A disintegrin-like and metalloprotease domain with thrombospondin type 1 motifs 9 (ADAMTS9) is closely related to lymph node metastases, and it can inhibit tumor growth by suppressing angiogenesis in NPC.^10^, ^11^ The transcription factor (TF) adaptor-related protein complex 1 (AP-1) activated by the EBV-encoded Nuclear Antigen 1 (EBNA1) can target hypoxia-inducible factor-1α, interleukin 8 and Vascular Endothelial Growth Factor (VEGF), which promotes microtubule formation in NPC cells.^12^ The TF Forkhead Box M1 (FOXM1) is involved in tumor development, and the adenovirus vector AdFOXM1shRNA, which expresses FOXM1-specific short hairpin RNA, may be used as a therapeutic intervention for the treatment of patients with NPC.^13^ By down-regulating the expression of Secreted Protein, Acidic and Rich in Cysteine (SPARC), TF sex determining region Y-box 5 (SOX-5) functions in the progression of NPC and may be used as a predictor for poor NPC prognosis.^14^, ^15^ Although these studies have been performed to investigate NPC, the mechanisms of NPC still remain unclear.

In 2006, Sengupta et al. analyzed the expression of all latent EBV genes between NPC samples and normal nasopharyngeal epithelium samples, and obtained a panel of differentially expressed genes (DEGs).^3^ Using the same data that was used by Sengupta et al.,^3^ we not only screened the DEGs but also performed a comprehensive bioinformatic analysis to identify key genes associated with NPC. The potential functions of the DEGs were predicted by functional and pathway enrichment analyses. In addition, a TF-DEG regulatory network was constructed to investigate the regulatory relationships between TFs and DEGs.

## Methods

### Microarray data

The GSE12452 expression profile data deposited by Sengupta et al.^3^ was downloaded from the Gene Expression Omnibus (GEO, http://www.ncbi.nlm.nih.gov/geo/) database, which was based on the platform of the GPL570 [HG-U133_Plus_2] Affymetrix Human Genome U133 Plus 2.0 Array. The specimens included 31 NPC samples and 10 normal nasopharyngeal tissue samples collected from patients from Taiwan with informed consent. The samples were resected, fast frozen and then stored in liquid nitrogen.

### Screening for DEGs

For the GSE12452 dataset, the Robust MultiArray Averaging (RMA) method in the Partek^®^ Genomics Suite™ (PGS) (http://www.partek.com/) package^16^ was used for preprocessing, including background adjustment, quantile normalization and log 2 transformation. After batch correction, the processed expression matrix was obtained. The ANOVA (analysis of variance) method in the PGS package^16^ was used to compare the DEGs between the NPC samples and the normal nasopharyngeal tissue samples. The *p*-values obtained were adjusted for multiple testing using the Benjamini and Hochberg method.^17^ Genes with fold-change (FC) >2 and adjusted *p*-value < 0.05 were selected as DEGs.

### Functional and pathway enrichment analysis

Gene Ontology (GO) terms can be used to describe three types of gene products including biological process in which they implicated, their molecular function and subcellular location.^18^ The Kyoto Encyclopedia of Genes and Genomes (KEGG) is a database composed of genes and their functional information.^19^ Using the BiNGO plugin^20^ in Cytoscape (http://www.cytoscape.org/) and pathway enrichment analysis in the PGS package,^16^ GO and KEGG pathway enrichment analyses were performed separately for DEGs between the NPC samples and the normal nasopharyngeal tissue samples. The adjusted *p*-value < 0.05 was used as the cut-off criterion.

#### TF-DEG regulatory network construction

The Genomatix Software Suite (https://www.genomatix.de/) package^21^ was used to predict Transcription Factors (TFs). In brief, the Gene2Promoter (http://www.genomatix.de) tool^22^ was used to extract promoter sequences of corresponding DEGs from the Genomatix database. Then, the TFs were analyzed using the MatInspector tool.^21^ TFs with a *p*-value of <0.05 and FC > 2 were selected as differentially expressed TFs, and then their corresponding TF-DEG pairs were screened. Finally, the TF-DEG regulatory network was visualized using Cytoscape software.^23^

## Results

### Analysis of DEGs

**Figure 1:**
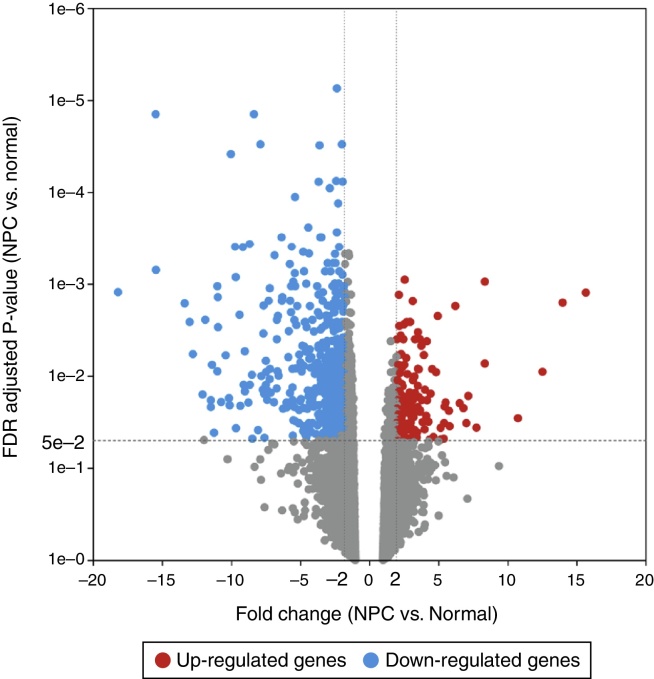
The volcano plot for differentially expressed genes (DEGs) (FC > 2 and adjusted *p*-value < 0.05). The horizontal axis represents the fold change, and the vertical axis represents the adjusted *p*-value. The red and blue circles indicate up- and down-regulated genes, respectively.

**Figure 2:**
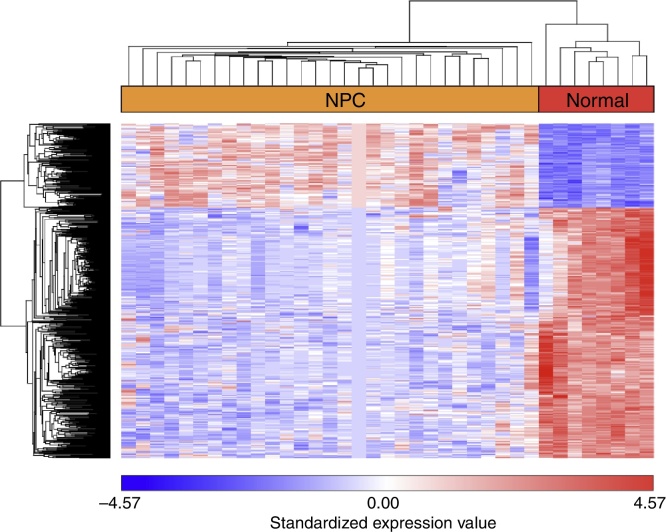
The heat map of the hierarchical cluster analysis for differentially expressed genes (DEGs).

After GSE12452 was downloaded, the expression profile data were preprocessed and then the DEGs were identified by ANOVA using the PGS package. The volcano plot for DEGs separately is shown in Fig. 1. A total of 487 genes were selected as DEGs between the NPC samples and the normal nasopharyngeal tissue samples, including 122 up-regulated and 365 down-regulated genes. The number of down-regulated genes was greater than the number of up-regulated genes. In the heat map of the hierarchical cluster analysis for the DEGs, the NPC samples and normal nasopharyngeal tissue samples were clearly divided into two groups (Fig. 2).

#### Functional and pathway enrichment analysis

The top 10 GO functions for the up-regulated genes included collagen fibril organization (*p* = 1.44E−06), extracellular matrix organization (*p* = 6.73E−08) and regulation of biological process (*p* = 3.35E−06, which involved prostaglandin-endoperoxide synthase 2, PTGS2) (Table 1A). The enriched KEGG pathways for the up-regulated genes are listed in Table 1C, and these included amoebiasis (*p* = 1.25E−07), ECM-receptor interaction (*p* = 9.01E−10) and small cell lung cancer (*p* = 3.10E−06, which involved PTGS2).

**Table 1:** The enriched GO functions and KEGG pathways for the up-regulated and the down-regulated genes. (A) The top 10 GO functions enriched for the up-regulated genes. (B) The top 10 GO functions enriched for the down-regulated genes. (C) The top 10 KEGG pathways enriched for the up-regulated genes. (D) The top 10 KEGG pathways enriched for the down-regulated genes.

Term	Description	Gene number	Gene symbol	*p*-Value
*A*				
30198	Extracellular matrix organization	9	COL18A1, COL4A2……	6.73E−08
30199	Collagen fibril organization	5	COL3A1, COL1A2……	1.44E−06
43062	Extracellular structure organization	9	COL3A1, COL1A2……	2.85E−06
50789	Regulation of biological process	72	PTGS2, EZH2……	3.35E−06
32964	Collagen biosynthetic process	3	COL1A1, COL5A1, COL5A1	3.91E−06
43588	Skin development	5	COL5A2, COL5A1……	4.77E−06
65007	Biological regulation	74	SLC9A7, PTGS2……	6.95E−06
6950	Response to stress	30	C3AR1, PTGS2……	8.65E−06
9611	Response to wounding	15	BMP2, TNFSF4……	1.03E−05
6954	Inflammatory response	11	CXCL9, PLA2G7……	2.15E−05

*B*				
10880	Regulation of release of sequestered calcium ion into cytosol by sarcoplasmic reticulum	4	CALM3, FKBP1B……	2.32E−07
30855	Epithelial cell differentiation	13	EREG, ELF3……	1.30E−06
60316	Positive regulation of ryanodine-sensitive calcium-release channel activity	3	CALM2, CALM1, CALM1	3.21E−06
60314	Regulation of ryanodine-sensitive calcium-release channel activity	4	FKBP1B, CALM2……	9.20E−06
60315	Negative regulation of ryanodine-sensitive calcium-release channel activity	3	CALM3, CALM2, CALM1	1.27E−05
7017	Microtubule-based process	14	TPPP3, DNAH9……	1.92E−05
8544	Epidermis development	12	CST6, EREG……	2.15E−05
1539	Ciliary or flagellar motility	4	TEKT2, DNAH2……	3.02E−05
7018	Microtubule-based movement	9	DNAH2, DNAH7……	3.72E−05
30216	Keratinocyte differentiation	7	PPL, CNFN……	3.90E−05

*C*				
	ECM-receptor interaction	11	COL1A1, COL1A2……	9.01E−10
	Protein digestion and absorption	10	COL1A2, COL3A1……	9.05E−09
	Amoebiasis	10	CD14, COL1A2……	1.25E−07
	Small cell lung cancer	8	PTGS2, FN1……	3.10E−06
	*Staphylococcus aureus* infection	5	C3AR1, FCGR2A……	2.66E−04
	Osteoclast differentiation	6	SOCS1, TYK2……	2.23E−03
	Toll-like receptor signaling pathway	5	CXCL9, NFKBIA……	4.54E−03
	DNA replication	3	PCNA, MCM2……	6.36E−03
	Leishmaniasis	4	FCGR2A, PTGS2……	6.94E−03
	Cell cycle	5	CCNE2, SMC1A……	9.53E−03

*D*				
	Drug metabolism-cytochrome P450	9	ADH1 C, ADH7……	3.02E−07
	Metabolism of xenobiotics by cytochrome P450	8	ADH7, ALDH1A3……	8.48E−06
	Tyrosine metabolism	5	ALDH3A1, ALDH3B2……	1.69E−04
	Glycolysis/gluconeogenesis	6	ADH7, ALDH1A3……	2.51E−04
	Histidine metabolism	4	ALDH3A1, ALDH3A2……	5.74E−04
	beta-Alanine metabolism	4	ALDH3A2, ALDH3B2……	6.59E−04
	Phenylalanine metabolism	3	ALDH1A3, ALDH3A1, ALDH3B2	1.88E−03
	Tight junction	7	CGN, CLDN7……	2.40E−03
	Phototransduction	3	CALM1, CALM2, CALM3	7.52E−03
	Retinol metabolism	4	DHRS9, SDR16C5……	1.03E−02

Similarly, functional and pathway enrichment analyses were performed for the down-regulated genes. As shown in Table 1B, the functions including the regulation of release of sequestered calcium ion into the cytosol by sarcoplasmic reticulum (*p* = 2.32E−07), epithelial cell differentiation (*p* = 1.30E−06) and positive regulation of ryanodine-sensitive calcium-release channel activity (*p* =  3.21E-06) were enriched. The enriched pathways included drug metabolism-cytochrome P450 (*p* = 3.02E−07), metabolism of xenobiotics by cytochrome P450 (*p* = 8.48E−06) and tyrosine metabolism (*p* = 1.69E−04) (Table 1D).

#### TF-DEG regulatory network analysis

**Figure 3:**
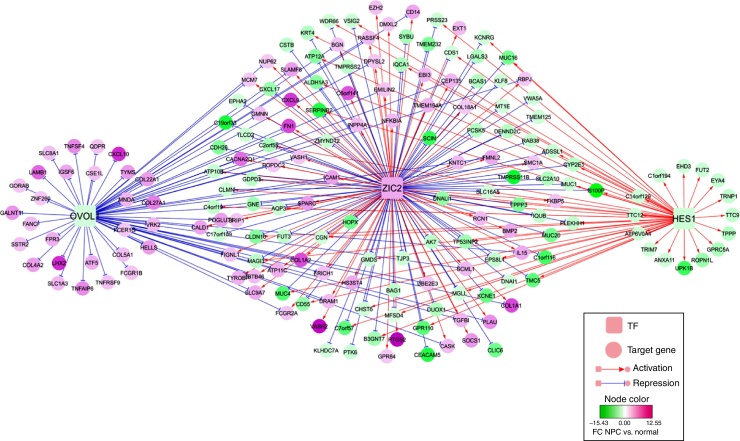
The regulatory network of Transcription Factors (TFs, such as ZIC2, OVOL and HES1) and their targeted differentially expressed genes (DEGs).

Using the Genomatix Software Suite package, TFs were predicted for the 122 up-regulated and 365 down-regulated genes. There were up-regulated TFs targeting up-regulated or down-regulated genes, as well as down-regulated TFs targeting up-regulated or down-regulated genes. However, only TFs that targeted both up-regulated and down-regulated genes were selected. The TF-DEG regulatory network of TFs (Zic family member 2, ZIC2; ovo-like 1, OVOL1; and hairy and enhancer of split 1, HES1) and their targeted DEGs are shown in Fig. 3. In particular, significantly up-regulated PTGS2, fibronectin (FN1) and chemokine (C-X-C motif) ligand 9 (CXCL9) were targeted and activated by ZIC2. Nevertheless, significantly up-regulated chemokine (C–X–C motif) ligand 10 (CXCL10) was targeted and suppressed by OVOL1.

## Discussion

In this study, a total of 487 genes were selected as DEGs between NPC samples and normal nasopharyngeal tissue samples, including 122 up-regulated and 365 down-regulated genes. There were many functions enriched for the DEGs, such as extracellular matrix organization and extracellular structure organization. PTGS2, FN1, CXCL9 and CXCL10 were significantly up-regulated genes involved in the TF-DEG regulatory network, and they may function in NPC through ZIC2 or OVOL1 (e.g., ZIC2→PTGS2 and OVOL1→CXCL10).

Enrichment analysis indicated that PTGS2 was involved in the regulation of biological process and small cell lung cancer. As a downstream gene involved in the NF-κB pathway, PTGS2 is also referred to as cyclooxygenase-2 (COX-2). It is known that COX2 can regulate cancer stem-like cells of NPC cells and promote their characteristics. Furthermore, parthenolide may function in targeted chemotherapy via the NF-κB/COX2 pathway.^24^ Based on PCR and restriction fragment length polymorphism analysis, the COX2 −765 G>C functional promoter polymorphism may be related to risk and neoplastic progression of NPC.^25^ The results of immunohistochemistry and semiquantitative assessment show that COX-2 expression increases as the nasopharyngeal epithelium develops from normal to dysplastic and then into NPC, indicating that COX-2 promotes the development of NPC.^26^, ^27^ This suggests that PTGS2 might be associated with NPC.

It is known that FN1 contributes to tumorigenesis by promoting the growth and migration of tumor cells, as well as by enhancing resistance to therapy.^28^, ^29^ Up-regulated FN1 correlates with the epithelial to mesenchymal transition (EMT) and contributes to tumor cell metastasis; therefore, it may be used as a potential diagnostic marker and therapeutic target of NPC.^30^ Based on a multiplex suspension array system, a previous study found that CXCL9 expression is significantly up-regulated in patients with NPC and oral cavity squamous cell carcinoma.^31^, ^32^ Quantitative real-time PCR and immunohistochemistry suggested that up-regulated CXCL9 is related to the aggressiveness of NPC, and an enzyme-linked immunosorbent assay indicated that its serum level may be a valuable prognostic indicator.^33^ The ZIC genes, which consist of five Cys2His2 zinc-finger domains, encode zinc-finger Transcription Factors.^34^ Among the family members, ZIC2 mediates the tissue-specific expression of the G-protein coupled receptor dopamine receptor D1.^35^ ZIC2 expression is up-regulated in several malignant tumors, including synovial sarcoma, pediatric medulloblastoma and endometrial cancers.^36^, ^37^, ^38^ Therefore, the expression levels of FN1, CXCL9 and ZIC2 might be correlated with NPC. In the TF-DEG regulatory network, we found that ZIC2 targeted and activated FN1, CXCL9 and PTGS2, indicating that ZIC2 might also function in NPC by regulating FN1, CXCL9 and PTGS2.

It is reported that the TFs OVOL1 and OVOL2 play critical roles in inducing the transition from mesenchymal to epithelial (MET) in several types of human cancers.^39^ As a member of the CXC chemokine family, CXCL10 binds to its receptor CXCR3 to exert biological functions in infectious diseases, immune dysfunction, chronic inflammation, tumor development and metastasis.^40^ The ELR-negative CXC chemokine CXCL10 can attenuate angiogenesis and suppress tumors.^41^ Overexpression of CXCL10 and CXCR3 play essential roles in advanced cancers, such as basal cell carcinoma,^42^ B-cell lymphoma,^43^ multiple myeloma^44^ and ovarian carcinoma.^45^ Inflammatory cytokines including CXCL10, Macrophage-Inhibitory-Protein 1 (MIP1) and interleukin 1 alpha are overexpressed in malignant NPC cells, which may promote the leukocyte infiltrate.^46^ These results suggest that OVOL1 and CXCL10 might be correlated with NPC. In the TF-DEG regulatory network, we also found that OVOL1 targeted and repressed CXCL10, suggesting that OVOL1 might also play a role in NPC by mediating CXCL10.

## Conclusion

In summary, we used GSE12452 expression profile data downloaded from GEO to study the mechanisms of NPC. A total of 487 genes were selected as DEGs between the NPC samples and the normal nasopharyngeal tissue samples. Several genes (PTGS2, FN1, CXCL9, CXCL10, ZIC2 and OVOL1) might play roles in NPC and might also be used for targeted therapy of NPC in clinical practice. However, further experimental validation is still required to unravel their mechanisms of action in NPC.

## Conflicts of interest

The authors declare no conflicts of interest.
